# Emergency department utilization for sickle cell disease in St. Vincent and the Grenadines

**DOI:** 10.11604/pamj.2021.38.100.27424

**Published:** 2021-01-29

**Authors:** Shelly-Ann Williams, Shakel Henson, Shenese Trimmingham, Jill Newman, Julie Kanter

**Affiliations:** 1 Department of Pediatrics, Medical University of South Carolina, Charleston, South Carolina, United States of America,; 2 Department of Histology, American University of St. Vincent School of Medicine, St. Vincent and the Grenadines, West Indies,; 3 Department of Medicine, University of Alabama at Birmingham, Birmingham, Alabama, United States of America

**Keywords:** Sickle cell disease, St. Vincent, Grenadines, diagnosis, emergency service, emergency department

## Abstract

**Introduction:**

sickle cell disease (SCD) is a chronic illness. Individuals affected by this disease are at risk for lifelong complications including episodes of acute pain, chronic pain and multi-organ injury that leads to reduced quality of life and a shortened life span. There is a wealth of data on acute care utilization for SCD in the United States. However, data from the Caribbean region is limited. The objective of this study is to explore Emergency Department (ED) utilization for SCD in St. Vincent and the Grenadines by describing: i) the characteristics of SCD related ED encounters; ii) the urgency of these encounters as defined by resource utilization; iii) the disposition for these ED encounters.

**Methods:**

the study was a cross-sectional study utilizing data from the ED log books at the Milton Cato Memorial Hospital (MCMH) during non-consecutive time periods between January 1^st^, 2012 - December 31^st^, 2016.

**Results:**

there were 666 SCD-related ED encounters during the study period. Thirty-four percent of encounters resulted in hospitalization and 66% of encounters met criteria for an urgent visit. The most commonly reported diagnosis was vaso-occlusive crisis and accounted for 84% of all encounters. The most frequently documented age group was the 18-30 age category at 43%.

**Conclusion:**

although SCD comprised less than 2% of all ED visits, the majority of these visits could be classified as urgent visits based on resource utilization. This study adds to the emerging data on the burden of this disease in this St. Vincent and the Grenadines.

## Introduction

Sickle cell disease (SCD) is a chronic illness that is caused by a mutation in the beta globin chain of the hemoglobin molecule [1]. Individuals affected by this disease are at risk for long life complications including episodes of acute pain, chronic pain and multi-organ injury that leads to reduced quality of life and a shortened life span [1,2]. Studies from the United States (US) have shown that individuals with SCD have high rates of acute care utilization [3,4]. Data have also shown that individuals >18 years of age and uninsured or publicly insured have the highest acute care utilization rates [3,5,6]. In the absence of national SCD registries, it can be difficult to describe the burden of disease in populations. As such, researchers have relied on other approaches such as healthcare utilization data to help in identifying the needs of individuals with SCD [6]. Although there are concerns that high utilization rates may be due to inappropriate use of acute care services, some data support that these high rates may not be preventable and may in-fact be due to the severity of the illness [7,8]. Therefore, emergency department (ED) utilization studies may still be able to provide meaningful data regarding the burden of disease in populations. There is a wealth of data on acute care utilization for SCD in the US. However, data from the Caribbean region is limited. This study aims to evaluate ED use for SCD in St. Vincent and the Grenadines. St. Vincent and the Grenadines is a multi-island nation in the Caribbean where it is estimated that 1 in 172 newborns may be affected by SCD [9,10]. The objective of this study is to explore emergency department (ED) utilization for SCD in St. Vincent and the Grenadines by describing; i) the characteristics of SCD related ED encounters; ii) the urgency of these encounters as defined by resource utilization; iii) the disposition for these ED encounters.

## Methods

**Data source:** the study was a cross-sectional study utilizing data from the ED log books at the Milton Cato Memorial Hospital (MCMH). Milton Cato Memorial Hospital was the country´s only public secondary referral hospital during the study period. At present, there is no computerized database of ED encounters at this hospital. Instead, there are hand-written logbooks that detail all ED encounters. For each ED encounter, the patient´s name, sex, age (but not date of birth), address, diagnoses, interventions performed during the encounter and final disposition are entered into logbooks. These logbooks provided the data for this study.

**Study period and study population:** the initial plan was to review all consecutive ED encounters from January 1^st^, 2012 - December 31^st^, 2016 and extract all SCD-related encounters. However, not all logbooks for this time period could be located. As a result, the data was limited to the non-consecutive block periods of 1/19/2012 -5/14/2012, 9/14/2013 - 12/31/2013, 1/1/2014 - 3/31/2014, 1/10/2015 - 12/31/2015 and 1/1/2016 - 1/28/2016. All ED encounters for the time periods that were available were manually reviewed and SCD-related encounters were extracted. As there are no ICD codes documented in this logbook, all encounters with SCD or a SCD-related diagnosis were manually extracted and classified using the following criteria.

**A confirmed SCD related encounter was defined as:** i) an encounter that included documentation of SCD in the list of diagnoses OR; ii) an encounter with a SCD specific diagnosis (acute chest syndrome, vaso-occlusive crisis) without a documentation of SCD.

**A possible SCD related encounter was defined as:** an encounter that included an implied diagnosis, without a documentation of SCD (e.g. pain crisis, “in crisis”), confirmed and possible SCD related encounters were included in the final analyses.

**Variables:** for eligible encounters we collected patient age at time of encounter, address, sex, date of the encounter, diagnoses documented, interventions performed and the final disposition. Study data were extracted from the logbooks and managed using Research Electronic Data Capture (REDCAP) tools hosted at the Medical University of South Carolina [11]. Research electronic data capture (REDCAP) is a secure, web-based software platform designed to support data capture for research studies, providing: 1) an intuitive interface for validated data capture; 2) audit trails for tracking data manipulation and export procedures; 3) automated export procedures for seamless data downloads to common statistical packages; and 4) procedures for data integration and interoperability with external sources.

**Urgency determination:** resource utilization has been used in pediatric literature to determine the urgency of an ED encounter. Previous studies have defined an urgent ED encounter as any encounter that i) results in an admission or ii) required an ED based service such as a laboratory test, a radiographic study, an electrocardiogram or an echocardiogram [7,12,13]. Echocardiography was not available at this institution during the study period. Therefore, for this study, we defined an urgent encounter as any ED encounter that resulted in an admission OR a “treat and release” encounter that included any laboratory testing or any radiographic study.

**Statistical analysis and outcomes:** descriptive analyses were performed using the SAS version 9.4 (Cary, USA). Frequencies and proportions were used to describe study demographics, population clinical characteristics and diagnoses. Diagnoses, encounter classifications and encounter dispositions were further analyzed by age groups and reported as frequencies and percentages.

## Results

**Figure 1:**
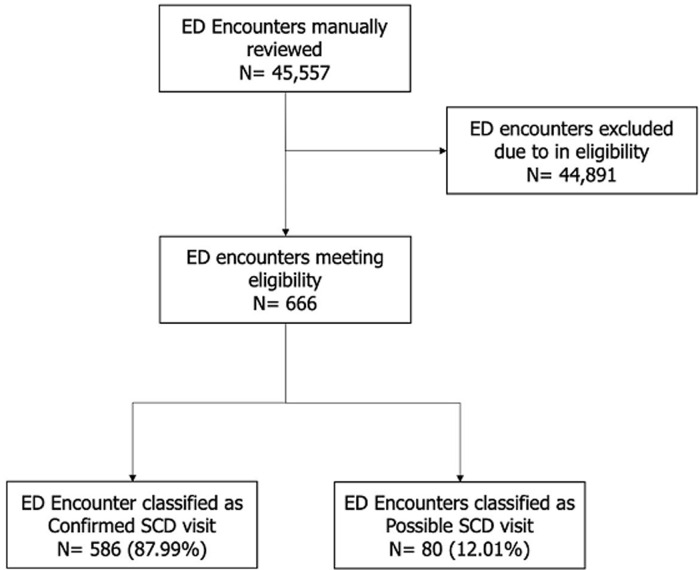
flow diagram of study population

During the periods for which data were available, there were a total of 45,557 visits to the ED. Of these 666 (1.46%) met eligibility criteria and were included in the final analysis. Eighty-eight percent were classified as confirmed SCD-related encounters and 12% were classified as a possible SCD-related encounter (Figure 1). Patient and ED encounters are summarized in (Table 1). Sixty-seven percent of the encounters were by female patients. The age 18-30 category had the most frequent encounters at 42.94% followed by the age category of 10-17 at 20.42%. Among the diagnoses, vaso-occlusive crisis (VOC) was the most commonly recorded diagnosis at 83.63%. Acute chest syndrome (7.36%) and acute viral illness (3.30%) were the second and third most commonly recorded diagnosis respectively. Thirty-four percent of encounters resulted in hospitalization and 66% of encounters met criteria for an urgent visit. Table 2 stratifies diagnoses by age. Across all age groups, VOC was the most commonly recorded diagnosis. Although there were some differences in the second and third most commonly recorded diagnosis among the different groups, lung conditions in the form of either acute chest syndrome or pneumonia/lower respiratory tract infections were in the top three most commonly reported diagnoses across all age groups. Encounter classification and disposition were also evaluated and stratified by age and are summarized in (Table 3, Table 4). For all age groups, the majority of encounters were classified as an urgent visit.

**Table 1:** characteristics of ED encounters for SCD from 2012-2016 (n=666)

Encounter characteristics	Number (percent)
**Gender**	
Male	218 (32.73)
Female	447 (67.12)
Not listed	1 (0.15)
**Age of Patient (years)**	
0-9	78 (11.71)
10-17	136 (20.42)
18-30	286 (42.94)
31-45	133 (19.97)
46-64	30 (4.50)
>65	3 (0.45)
**Visit disposition**	
Encounters ending in hospitalizations	228 (34.23)
Encounters ending in treat and release	371 (55.71)
Encounters ending with “Left without being seen” or “Left against medical advice”	10 (1.5)
Not listed	57 (8.56)
**Visit classification**	
Urgent	440 (66.07)
Non-urgent	226 (33.93)
**Diagnoses**	
Vaso-occlusive crisis	557 (83.63)
Acute chest syndrome	49 (7.36)
Acute viral illness	22 (3.30)
Pneumonia/lower respiratory tract infection	17 (2.55)
Urinary tract infection	17 (2.55)
Anemia	16 (2.40)
Pregnancy	13 (1.95)
Upper respiratory tract infection	12 (1.80)
Leg Ulcers/Skin, bone and soft tissue infections	11 (1.65)
Gastritis/gastroesophageal disease	10 (1.50)
Acute gastroenteritis	8 (1.20)
Asthma/wheezing	6 (0.90)
Fever/pyrexia	2 (0.30)
Priapism	1 (0.15)

**Table 2:** diagnoses by age groups (n=666)

Diagnosis	0-9	10-17	18-30	31-45	46-64	>65
Vaso-occlusive crisis	60 (76.92)	113 (83.09)	244 (85.31)	113 (84.96)	24 (80.00)	3 (100)
Acute chest syndrome	3 (3.85)	10 (7.35)	22 (7.69)	4 (3.01)	9 (30.00)	1 (33.33)
Acute viral illness	7 (8.97)	6 (4.41)	1 (0.35)	8 (6.02)	0 (0.00)	0 (0.00)
Pneumonia/lower respiratory tract infection	9 (11.54)	4 (2.94)	9 (3.15)	3 (2.26)	1 (3.33)	1 (33.33)
Urinary tract infection	1 (1.28)	2 (1.47)	10 (3.50)	4 (3.01)	0 (0.00)	0 (0.00)
Anemia	1 (1.28)	5 (3.68)	6 (2.10)	0 (0.00)	4 (13.33)	0 (0.00)
Pregnancy	0 (0.00)	0 (0.00)	12 (4.20)	1 (0.75)	0 (0.00)	0 (0.00)
Upper respiratory tract infection	3 (3.85)	2 (1.47)	4 (1.40)	2 (1.50)	1 (3.33)	0 (0.00)
Leg ulcers/ Skin, bone and soft tissue infections	2 (2.56)	1 (0.74)	6 (2.10)	2 (1.50)	0 (0.00)	0 (0.00)
Gastritis/gastroesophageal reflux disease	2 (2.56)	2 (1.47)	4 (1.40)	2 (1.50)	0 (0.00)	0 (0.00)
Acute gastroenteritis	1 (1.28)	1 (0.74)	2 (0.70)	2 (1.50)	0 (0.00)	2 (66.67)
Asthma/wheezing/ bronchial asthma	1 (1.28)	2 (1.47)	3 (1.05)	0 (0.00)	0 (0.00)	0 (0.00)
Fever/ pyrexia	2 (2.56)	0 (0.00)	0 (0.00)	0 (0.00)	0 (0.00)	0 (0.00)
Priapism	0 (0.00)	0 (0.00)	0 (0.00)	1 (0.75)	0 (0.00)	0 (0.00)

**Table 3:** encounter classification by age groups (n=666)

Encounter classification	0-9	10-17	18-30	31-45	46-64	>65
Urgent visit	56 (71.79)	100 (73.53)	178 (62.24)	82 (61.65)	21 (70.00)	3 (100)
Non-urgent visit	22 (28.21)	36(26.47)	108 (37.76)	51(38.35)	9 (30.00)	0 (0.00)

**Table 4:** encounter disposition by age groups (n=666)

Encounter disposition	0-9	10-17	18-30	31-45	46-64	>65
Admission	36 (48.65)	49 (39.20)	100 (39.53)	32 (26.89)	10 (40.00)	1 (33.33)
Treat and release	38 (51.35)	76 (60.80)	153 (60.47)	87 (73.11)	15 (60.00)	2 (66.67)

## Discussion

This study utilized available national level data to describe ED utilization for SCD in St. Vincent and the Grenadines. Results of this study showed that less than 2% of all ED encounters were SCD related encounters. However, the majority of encounters could be classified as urgent visits. Similar to other published literature, VOC accounted for the majority of SCD-related encounters [14-16]. When stratified by age, VOC still remained the most common diagnosis. The most frequently documented age group was the 18-30 age category, which is also similar to data from Schlenz *et al*. [6]. About 1/3 of all encounters resulted in an admission, which is similar to other studies that showed admission rates of around 29% [14]. Although for all age groups most encounters resulted in discharge, when looked at using resource utilization, most encounters were classified as urgent encounters. To our knowledge, this the first study that examines ED utilization on a national level for St. Vincent and the Grenadines. This study therefore provides valuable insight that will likely benefit health officials and health care providers in St. Vincent and the Grenadines. This study has several limitations. First, we were unable to identify unique individuals given the limitations of the data that are stored in the logbooks. As a result, we were not able to provide data on acute care utilization rates per individual.

Data have shown that in any given year, 50-60% of persons with SCD have no ED visits but that 20% of patients will have 3 or more acute care encounters per year [3,4,17]. Therefore, it is possible that there may have been a small subset of “high utilizers” that may be driving the results of this study. Nevertheless, this study is the first to provide national estimates for ED utilization for SCD in St. Vincent and the Grenadines. Since ED services are publicly funded in this population, these data will be very beneficial and informative to local health officials. The second major limitation was that many of the logbooks could not be located. As such, our data was limited to non-consecutive time periods between January 1^st^, 2012 to December 31^st^, 2016. It is possible that this could have affected the results of our study. However, the missing logbooks would have included all ED visits for those missing time periods. Therefore, non SCD-related encounters would have been missing as well. Given the number of SCD related encounters (n=666) that were available for analysis, we believe that this is a large enough sample size to provide meaningful results. Third, it was not always possible to determine the primary diagnosis for the encounters based on the information that was entered into the logbooks. It is possible that in some cases, SCD was a secondary diagnosis and not related to the reason for the visit. Nonetheless, our results revealed that VOC crisis was the most commonly documented diagnosis. This is in keeping with other published data suggesting that we were still able to provide a reasonable description of ED utilization for SCD in this population [14,15,18]. Finally, the total population of individuals living with SCD in this country is not known. Thus, it is unclear the percentage of individuals that visit the ED. Future studies should be conducted that address utilization at the individual level.

## Conclusion

Individuals with SCD utilize the ED for a variety of reasons. The most frequently documented diagnosis was VOC, and the age group 18-30 was the most commonly documented age group. Although SCD comprised less than 2% of all ED visits, the majority of these visits could be classified as urgent visits based on resource utilization. This study adds to the emerging data on the burden of this disease in this St. Vincent and the Grenadines. It will likely inform local providers and policy makers on the ED utilization for SCD and can lead to improvements in care provided to individuals with SCD in the future.

### What is known about this topic

- Individuals with sickle cell disease have high acute care utilization rates sickle cell disease;
- Data from US shows that adults >18 years of age or uninsured show highest acute care utilization rates.

### What this study adds

- There is currently limited data on acute care utilization for sickle cell disease in the Caribbean;
- This is the first study to evaluate acute care utilization for sickle cell disease in St. Vincent and the Grenadines.
